# Survival and Behavior of Potential Emerging Foodborne Pathogen *Streptococcus gallolyticus* subsp. *gallolyticus* During Manufacturing and Shelf Life of Kashar Cheese

**DOI:** 10.1002/fsn3.72131

**Published:** 2026-07-20

**Authors:** Müge Kayapınar, Nazmiye Ülkü Tüzemen, Artun Yıbar, Cüneyt Özakın, Recep Çıbık

**Affiliations:** ^1^ Department of Food Hygiene and Technology Institute of Health Sciences, Bursa Uludag University Bursa Türkiye; ^2^ Department of Medical Microbiology Faculty of Medicine, Bursa Uludag University Bursa Türkiye; ^3^ Department of Food Hygiene and Technology Faculty of Veterinary Medicine, Bursa Uludag University Bursa Türkiye

**Keywords:** cooked‐curd, food safety, Kashar Cheese, MALDI‐TOF‐MS, physicochemical analysis, *Streptococcus gallolyticus* subsp. *gallolyticus*, zoonotic foodborne pathogens

## Abstract

Members of the *Streptococcus bovis*/*Streptococcus equinus* complex (SBSEC), frequently detected in the microbiota of artisanal cheeses and as commensals in the gastrointestinal tracts of humans and animals, have raised concerns regarding their potential pathogenicity and the role of foodstuffs in transmission. *Streptococcus gallolyticus* subsp. *gallolyticus* (*Sgg*), associated with endocarditis and colorectal cancer, is a key member of this complex. This study aimed to investigate the survival and behavior of *Sgg* during the production and shelf life of Kashar Cheese. In sterilized milk used as a baseline growth model, *Sgg* exhibited biphasic growth, reaching levels above 11 log_10_ cfu/mL. Isolates were not able to survive in simulated gastric acidity conditions. The survival and behavior of *Sgg* during processing and storage of Kashar Cheese (a curd scalded Turkish Cheese) inoculated with *Sgg* at concentrations of 10^6^–10^8^ cfu/mL were monitored using traditional culturing, MALDI‐TOF‐MS and PCR analysis. Bacterial dynamics were tracked at sequential sampling times representing critical manufacturing and ripening stages: (a) time of inoculation, (b) before curd shearing, (c) curd before scalding, (d) after scalding, and throughout storage on days (e) 1, (f) 7, (g) 15, and (h) 30. Fermentative acidification by using yogurt cultures was more effective in reducing *Sgg* counts by day 30 of storage compared to lactic acid acidification, with mean reductions of 4.1 log_10_ and 1.0 log_10_, respectively; however, no statistically significant differences were observed among the groups (*p* > 0.05). The scalding step at 75°C for 5 min reduced the bacterial load substantially (3.3–4.1 log_10_), but did not achieve complete elimination. By day 30, *Sgg* remained detectable in cheeses produced with lactic acid (7.31 and 2.20 log_10_ cfu/g) and yogurt cultures (4.11 log_10_ cfu/g). These results indicate that while fermentative acidification by yogurt cultures provides better control over *Sgg* than lactic acid acidification, neither the starter cultures nor the 75°C scalding process is sufficient to completely eliminate this pathogen. Consequently, *Sgg* can successfully withstand traditional manufacturing and thermal hurdles, providing the first evidence of its survival within a cheese matrix during the ripening period. These findings highlight that artisanal or industrial curd‐scalded dairy products could serve as a potential vehicle for foodborne transmission of this pathogen, posing a genuine risk to public health. The survival of *Sgg* during Kashar Cheese production and storage was characterized. These findings highlight the potential for food‐chain transmission of *Sgg*. The results offer critical data to support improved food safety control measures.

## Graphical abstract

The experimental design and survival dynamics of *Streptococcus gallolyticus* subsp. g*allolyticus* (*Sgg*) in Kashar cheese. The right panel demonstrates the baseline growth characterization of *Sgg* strains in milk, as well as the inoculation of these strains into the process milk followed by traditional Kashar Cheese manufacturing across eight sequential sampling stages (a–h). The central panel highlights that even the critical curd‐scalding step at 75°C fails to achieve complete elimination, allowing the pathogen to persist throughout the ripening period. Consequently, the left panel illustrates the core public health concern: *Sgg* successfully withstands manufacturing hurdles and persists in the cheese matrix until Day 30 of storage, highlighting that consumption of contaminated cooked‐curd artisanal cheeses may serve as a potential carrier for human gastrointestinal colonization and subsequent systemic risks.

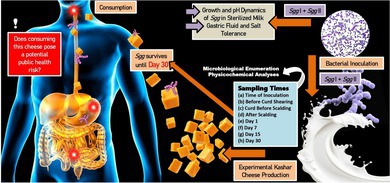

## 1 Introduction

The *Streptococcus bovis* / *Streptococcus equinus* complex (SBSEC), typically classified as Lancefield Group D, comprises Gram‐positive, catalase‐negative, non‐beta‐hemolytic cocci (Schlegel et al. 2003). The SBSEC group first drew attention in the early 1900s through the identification of streptococci from horses and cattle (Schlegel et al. 2003). Classical identification methods were soon found to be insufficient, and modern molecular approaches have since revealed a close link between SBSEC members and colorectal cancer (CRC), various human infections, as well as their frequent detection in food products (Kaindi et al. 2018; Putnam and Youn 2023). However, the safety of high‐level dietary intake of these bacteria remains uncertain (Kaindi et al. 2018), underlining the need to better understand their survival and pathogenic potential (Rusniok et al. 2010; Taylor et al. 2023).

Currently, seven subspecies are recognized within this complex: *S. gallolyticus* subsp. *gallolyticus* (*Sgg*), *S. gallolyticus* subsp. *macedonicus* (*Sgm*), *S. gallolyticus* subsp. *pasteurianus* (*Sgp*), *S. infantarius* subsp. *infantarius* (*Sii*), *S. lutetiensis* , *S. alactolyticus* , and *S. equinus* (Jans et al. 2015). Among them, *Sgg*, previously known as *S. bovis* biotype I, is notably associated with infective endocarditis (IE) and CRC (Ouranos et al. 2023). Other subspecies, such as *Sgm*, *Sii*, and *Sgp*, are more frequently detected in foods and have been linked to conditions including bacteremia and hepatobiliary diseases in adults, as well as neonatal meningitis (Kasamatsu et al. 2022).

Recent studies have highlighted the widespread occurrence of SBSEC members in fermented dairy products and traditional foods worldwide (Ayanoglu 2021; Chebeňová‐Turcovská et al. 2011; Mukisa et al. 2012). *Sgg*, although mainly recognized for its clinical significance, has also been isolated from various food matrices, raising important questions about its persistence and viability during food processing and storage (Jans and Boleij 2018). This dual presence makes it crucial to assess whether certain SBSEC members, including *Sgg*, can survive in foods such as cheese and what risks they might pose to consumers.

The frequent detection of these bacteria in dairy products is likely facilitated by their natural presence in the gastrointestinal tracts of dairy animals and their ability to withstand heat and salt stress during processing. Genomic analyses reveal that *Sgg* possesses adaptive traits for survival, including bile salt resistance, polysaccharide degradation, capsule formation, and biofilm development in the gut and food environments (Brouwer et al. 2016; Rusniok et al. 2010; Sillanpää et al. 2009). These features also help explain how *Sgg* may persist throughout the production and shelf life of dairy products.

Moreover, the zoonotic potential of *Sgg* cannot be overlooked, as human intestinal prevalence rates vary widely, and close contact with animals increases the risk of colonization (Dumke et al. 2017; Jans and Boleij 2018). In a case–control study, *Sgg* was detected in 62.5% of fecal samples from healthy volunteers, and colonization was significantly associated with close animal contact and the use of animal waste as fertilizer (Dumke et al. 2017). Beyond colonization, clinical studies have demonstrated that *Sgg* bacteremia and IE are frequently accompanied by underlying colorectal neoplasia, with reported co‐occurrence rates up to 60%–70% (Boleij and Tjalsma 2013; Boltin et al. 2015). Certain strains promote tumor growth, degrade dietary tannins (compounds with anticancer activity), and express bile salt hydrolase, which may enhance their survival and colonization in the gastrointestinal tract (Oehmcke‐Hecht et al. 2020). These traits, along with its ability to adhere to colon cells, persist in tumor microenvironments, and produce gallocin, may also contribute to its stability in food matrices, highlighting the contamination risk from raw milk or production surfaces (Warner and Mehta 2024). Thus, the detection of *Sgg* in dairy products not only represents incidental contamination but also raises concern about a potential foodborne route of transmission, through which consumers may become colonized and subsequently face an increased risk of IE or CRC. Considering all this information, it remains unclear whether *Sgg* is the initiating factor for CRC. Although it remains unclear whether *Sgg* acts as the initiating factor for CRC, accumulating evidence indicates that it accelerates this process through newly clarified mechanisms.

In light of these considerations, the occurrence of *Sgg* in cheese products deserves special attention. Particularly in the context of Turkish Kashar Cheese, which is traditionally produced from raw cow's milk and classified as a ‘cooked‐curd’ cheese, the curd‐scalding step typically utilizes specific time–temperature combinations (75°C for 5 min) that are sufficient to cause a significant decrease in a broad range of microorganisms. Still, the behavior of *Sgg* under the heat treatment and dynamic conditions of Kashar Cheese making remains largely undefined.

To address these critical knowledge gaps, this study aimed to systematically evaluate the survival and behavior of clinical *Sgg* strains across four distinct objectives: (i) to determine their growth dynamics in baseline sterilized milk, (ii) to assess their resistance under simulated gastric acidity conditions, (iii) to evaluate their tolerance to salt stress, and (iv) to monitor their persistence throughout the manufacturing stages and a 30‐day ripening and storage period of Kashar Cheese. Using traditional culturing, MALDI‐TOF‐MS, and PCR analysis, this study seeks to provide concrete data on whether this potential pathogen can overcome traditional dairy processing hurdles and pose a food safety risk to consumers.

## 2 Materials and Methods

### 2.1 Procurement of Milk and *Sgg* Strains

The raw milk used in this study was obtained from the Research Farm of the Faculty of Veterinary Medicine, Bursa Uludag University (B.U.U.). Pasteurization was performed at the Milk Processing Unit of the Animal Health and Animal Production Research and Application Center, which is part of the Faculty of Veterinary Medicine. Prior to the production of fresh Kashar Cheese, the milk underwent microbiological and physicochemical analyses, and the absence of antibiotic residues was confirmed.

The *Sgg* I and II strains used in this study were selected from 20 clinical isolates obtained from the B.U.U. Faculty of Medicine Hospital. Suspect isolates initially identified via MALDI‐TOF‐MS were subjected to PCR assays targeting seven MLST gene regions (*aroE, glgB, nifS, p20, tkt, trpD*, and *uvrA*) for molecular confirmation (Jolley and Maiden 2010). These two specific strains were selected because they were the only isolates that yielded positive bands across all seven target regions, definitively confirming their identity, and they represented the classic clinical manifestations associated with *Sgg*. Specifically, *Sgg* I was isolated from the blood culture of a patient presenting with fever and a systolic murmur, clinically indicative of IE, while *Sgg* II was recovered from a CRC patient who developed hepatic encephalopathy post‐surgery. All clinical isolates were completely anonymized to ensure patient privacy.

### 2.2 Growth and pH Dynamics of *Sgg* in Sterilized Milk, Gastric Fluid, and Salt Tolerance

To evaluate the growth characteristics of *Sgg* strains in milk and their effect on pH, a cocktail containing equal proportions of both *Sgg* strains (*Sgg* I and *Sgg* II) was inoculated together into sterilized milk (autoclaved at 121°C for 15 min) at ~10^5^ cfu/mL. The inoculated milk samples were initially incubated at 37°C for the first 72 h (3 days) to simulate early fermentation, and subsequently transferred to +4°C for the remainder of the 180‐day storage period to mimic the extended ripening and cold storage of Kashar Cheese. Viable counts and pH were determined at 0, 6, 12 h, as well as on days 1, 2, 3, 4, 7, 15, 30, 90, and 180.

To test survival in gastric fluid, logarithmic phase cells of each *Sgg* strain were evaluated separately at concentrations of 10^8^ and 10^6^ cfu/mL. The individual strains were washed and exposed to synthetic gastric fluid. The gastric fluid was prepared in a buffer solution containing NaCl (2.05 g/L), KH_2_PO_4_ (0.60 g/L), CaCl_2_ (0.11 g/L), and KCl (0.37 g/L), and adjusted to pH 2.0–2.5 with 1 M HCl. The mixture was then autoclaved at 121°C for 15 min. After cooling, pepsin (0.0133 g/L) and lysozyme (0.01 g/L) were added (Bautista‐Gallego et al. 2013). They were incubated at 37°C on an orbital shaker (Nüve, Shaker SL 350, Turkiye) to mimic the peristaltic movements of the gastric system. Then, the viability was assessed by spreading on M17 agar (Biokar Diagnostics, France) at 0, 5, 10, 15, 30, 60, 90, and 180 min. pH was also monitored at each sampling time.

For salt tolerance, two *Sgg* strains were separately incubated in M17 broth supplemented with varying concentrations of NaCl (1% to 14% concentrations with 1% increments) at 37°C for 24 h (Beck et al. 2008).

All experiments were performed in triplicate.

### 2.3 Experimental Kashar Cheese Production and Bacterial Inoculation

**FIGURE 1:**
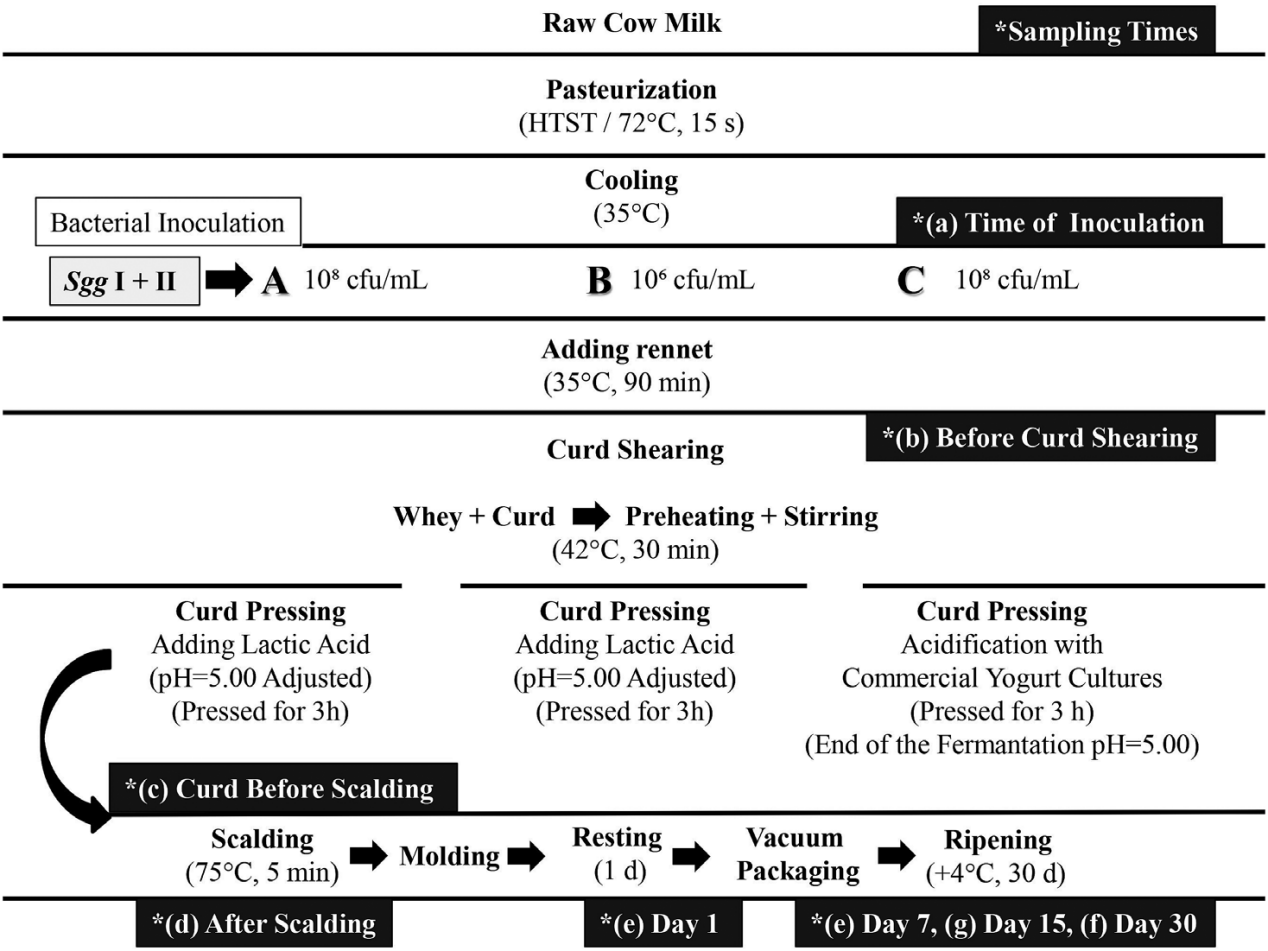
Flowchart of the cheese manufacturing process and sampling times.

Three groups were formed within the scope of the experiment. Each group was produced and evaluated in three replicates, resulting in a total of nine cheese blocks (*n* = 3 per group) from which separate analytical samples were collected at each scheduled sampling time point. In the first and second groups (A1–A2–A3 and B1–B2–B3), acidity was achieved with lactic acid (Alfasol, Turkiye), and *Sgg* was inoculated at 10^8^ and 10^6^ cfu/mL, respectively. In the third group (C1‐C2‐C3), acidity was achieved with commercial yogurt cultures (Danisco, France) containing *Lactobacillus delbrueckii* subsp. *bulgaricus* and *Streptococcus thermophilus* , and 10^8^ cfu/mL *Sgg* was added. Pasteurized milk was cooled to 35°C, the specified inoculation procedures were performed, and cheese production was then completed using conventional methods. Following inoculation, rennet was added to ensure coagulation, and the milk was allowed to coagulate for approximately 90 min until a firm curd was obtained. For the curd formation, the coagulum was cut. Then, the curd and whey mixture was preheated to 42°C with continuous stirring, which was achieved by adding hot sterile water, and this process lasted for 30 min. After the preheating stage, the curd was drained. In the group produced with yogurt cultures (C), acidification occurred biologically due to starter activity during a 3‐h fermentation stage, throughout which the pH was monitored until it naturally reached 5.0. In groups produced with lactic acid (A and B), no starter culture was used; therefore, the milk was acidified by direct lactic acid addition, to adjust the pH to 5.0 prior to the pressing stage and the curd was subsequently held under pressure for 3 h to synchronize the processing duration with Group C. This target pH of 5.0 is characteristic of Kashar Cheese manufacture and is essential for proper curd stretching and texture development. After the pressing/fermentation stage, the curd (at room temperature, ~22°C) was sliced into thin pieces (3–5 mm thickness), placed into a perforated basket, and scalded in a pasteurized brine containing 7% NaCl at an initial temperature of 75°C for 5 min, maintaining a curd‐to‐brine ratio of approximately 1:6 (w/v) (Ucuncu 2020). This procedure ensured that the internal temperature of the curd mass rapidly exceeded 70°C. Following the scalding and stretching process, the cheese was aged overnight in a ripening room, vacuum‐packaged, and matured at +4°C (Ucuncu 2020) (Figure 1).

The viability of *Sgg* during cheese production and ripening was measured at the time of inoculation, during curd shearing, before and after scalding, before packaging, and on days 7, 15, and 30. Physicochemical analyses were also performed at each of these sampling times (Figure 1).

### 2.4 Microbiological Enumeration of *Sgg*

Following the inoculation with *Sgg*, 1 mL of pasteurized milk was collected, serially diluted tenfold, and 100 μL from the appropriate dilutions were plated onto M17 agar plates. For cheese analysis, 5 g of sample was homogenized with 45 mL of Maximum Recovery Diluent (MRD) (Merck, Germany) using a stomacher (Seward, Stomacher 80 Lab Blender BA 6020, United Kingdom) for 30 s. Tenfold serial dilutions of the homogenate were prepared in MRD, and 100 μL of each selected dilution was plated onto M17 agar plates. All plates were incubated at 37°C for 24 ± 2 h, and cfu/mL‐g counts were determined for each sample on M17 agar, as recommended for the enumeration of lactic streptococci (Biokar Diagnostics, n.d.).

### 2.5 MALDI‐TOF‐MS Analysis and PCR Identification

For each group, three colonies (white to cream‐colored, round, with smooth edges) were selected from M17 agar plates at each sampling time for preliminary identification using the MALDI‐TOF‐MS system (Bruker Microflex LT, Bruker Daltonics, Germany). In total, over 216 colonies were subjected to MALDI‐TOF‐MS analysis throughout the study.

Following MALDI‐TOF‐MS analysis, ten colonies identified as *Sgg* were tested using conventional PCR, along with one negative control and two positive controls. Genomic DNA was extracted from all selected colonies using the NucleoSpin Microbial DNA Extraction Kit (Macherey‐Nagel, Düren, Germany) in accordance with the manufacturer's instructions. For molecular confirmation of the isolates, PCR assays targeting the seven housekeeping genes described in Section 2.1 were performed (Kumar et al. 2018; Jolley and Maiden 2010). The amplified DNA products were separated by agarose gel electrophoresis and visualized using a G:BOX Chemi XX9 imaging system (Syngene, Cambridge, United Kingdom). Isolates that yielded bands for all seven gene regions were considered positive for *Sgg*.

### 2.6 Physicochemical Analyses of Kashar Cheese Samples

Physicochemical analyses including pH, titratable acidity, water activity, moisture, dry matter, salt, and fat content were conducted on the cheese samples. All analyses were conducted in accordance with the official methods described by AOAC International (AOAC International 2019).

### 2.7 Statistical Analyses

All statistical analyses were performed using SPSS Statistics for Windows, Version 20.0 (IBM Corp., Armonk, NY, USA). Microbial counts (cfu/mL) were log_10_‐transformed prior to analysis to reduce skewness and stabilize variance; MALDI‐TOF‐MS colony identification results were evaluated as the proportion (%) of positive colonies; physicochemical data were analyzed directly.

Normality was assessed using the Shapiro–Wilk test and homogeneity of variances with Levene's test. As microbial enumeration data did not satisfy the assumptions of normality and variance homogeneity, nonparametric statistical tests were selected to ensure reliable interpretation. Accordingly, group comparisons were performed using the Kruskal–Wallis test. Where applicable, Dunn–Bonferroni or Tukey post hoc tests were applied.

Because initial inoculation levels differed by design among Groups A, B, and C, absolute microbial counts were not statistically compared across groups. Therefore, differences among groups were assessed based on percentage changes between processing stages, which also allowed a clearer depiction of temporal shifts in microbial counts. To evaluate intragroup temporal variations across the sequential sampling times under a repeated measures design, the Friedman test was used, followed by the Dunn‐Bonferroni post hoc test for pairwise comparisons. Statistically significant differences are indicated using lowercase superscript letters within the respective table columns. MALDI‐TOF‐MS data were analyzed for *Sgg* detection rates across Groups A, B, and C using the Friedman test for within‐group comparisons and the Kruskal–Wallis test for between‐group comparisons.

Statistical significance was set at *p* < 0.05.

## 3 Results and Discussion

### 3.1 Growth and pH Dynamics of *Sgg* in Sterilized Milk

**FIGURE 2:**
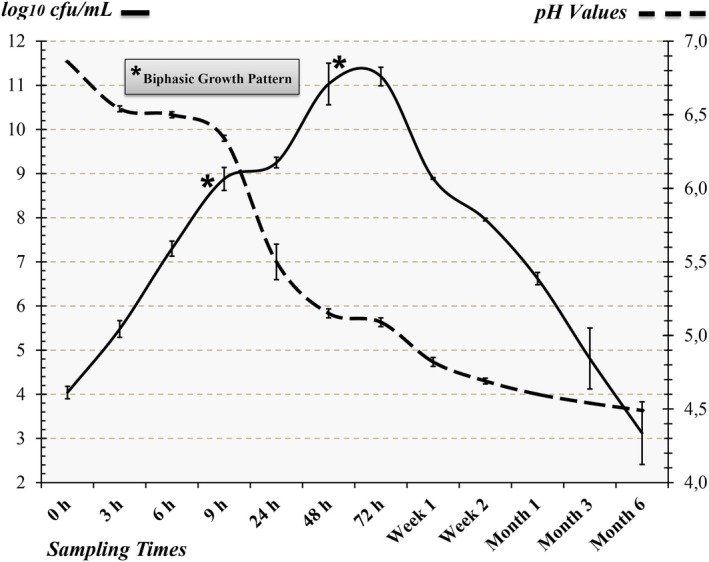
Time‐dependent changes in *Sgg* counts and pH values in sterilized milk showing a biphasic growth pattern.

The microorganism exhibited rapid growth in milk during the first 72 h, followed by a decline. Starting from an inoculum of ~10^4^–10^5^ cfu/mL, *Sgg* entered the logarithmic phase within 24 h and reached the late log phase by the 24th hour. By 48 and 72 h, the cultures attained 11.03 and 11.20 log_10_ cfu/mL, respectively, indicating the onset of a second rapid growth phase. This biphasic profile contrasts with the classical single‐phase model of bacterial growth (Figure 2). The viable count of bacteria decreased steadily after 72 h and reached 4.81 log_10_ cfu/mL by the end of the 3rd month.

Notably, *Sgg* strains reached higher concentrations than many lactic acid bacteria (LAB) typically adapted to dairy environments. For comparison, typical maximum growth of LAB strains such as *Lactococcus lactis* subsp. *cremoris* in milk has been reported to reach around 9 log_10_ cfu/mL (9.14 ± 0.03 after 48 h at 22°C) (Rodríguez et al. 2022), which is significantly lower than the maximum counts observed for *Sgg* in this study. In dairy substrates, LAB may exhibit either monophasic or biphasic growth patterns. Similarly to *Sgg*, *Lactobacillus kefiri* has been reported to exhibit biphasic growth in MRS medium, with the second phase associated with the recovery of energy from metabolic products, such as lactate (Kondybayev et al. 2022). Our experiments revealed that *Sgg* accelerated its growth again after 24 h, resulting in an additional increase of approximately 2 log units on a log_10_ basis. This may indicate that organic acids or degraded milk components (e.g., peptides, amino acids, or lactose derivatives) that accumulated in the medium could be used as secondary energy sources by the bacteria. These findings suggest that a clinical isolate of *Sgg* can readily grow in milk and therefore has high growth potential in dairy products such as cheese. A significant decrease in pH was observed during growth. The pH decreased from 6.86 to 4.49 (Figure 2). This decrease indicates that the bacteria can produce fermentative products such as lactic acid and other organic acids. Another noteworthy point is that, like many other LAB, *Sgg* remained viable at pH values below 5. This suggests that *Sgg*, with its high tolerance to acidic environments, may survive in acidic conditions found in foods. These characteristics also support *Sgg*'s ability to survive in the gastrointestinal tract after foodborne contamination and pose a potential infective risk to the host.

### 3.2 Tolerance of *Sgg* to Simulated Gastric Conditions

A rapid bactericidal effect was observed in both strains within the first 5 min of contact with the gastric environment. Control cultures at minute 0 confirmed viability, but growth was absent in all replicates for all subsequent time points. Experimental results indicate that *Sgg* I and *Sgg* II strains were unable to survive in synthetic gastric media at both concentrations. In particular, the rapid elimination of *Sgg* despite very high initial concentrations (10^8^ cfu/mL) also suggests that sensitivity to acid stress may vary by strain rather than by species. This indicates that *Sgg* may be highly inactivated during gastric transit, and therefore, different protective mechanisms (e.g., a protective food matrix, buffering agents) may be required for gastrointestinal colonization.

However, these findings do not eliminate the possibility that *Sgg* may cross the gastric barrier under certain physiological and dietary factors. One possible risk transmission scenario involves pharmacological or pathological suppression of gastric acidity. Proton pump inhibitors (PPIs) suppress gastric acidity by inhibiting the H^+^/K^+^ ATPase enzyme. Widespread use of these drugs (e.g., for the treatment of gastroesophageal reflux, gastritis, or ulcers) can raise gastric pH to levels of 4–6, providing a more favorable transit environment for many microorganisms (Janarthanan et al. 2012). Literature has shown that PPI use increases susceptibility to certain enteric infections (e.g., *Clostridium difficile* ) (Janarthanan et al. 2012). Bacteria, such as *Sgg*, which generally have low acid tolerance, can more easily complete gastric transit when the acid barrier is weakened. This is of clinical importance, particularly for *Sgg* strains associated with endocarditis and CRC.

Another pathway can result from physiological disorders of gastric acid secretion. Conditions such as hypochlorhydria, atrophic gastritis, and age‐related decreased gastric acid production similarly lead to decreased gastric acidity (Corcoran et al. 2007). In these individuals, intestinal colonization by microorganisms that are normally eliminated during gastric transit, such as *Sgg*, appears more likely.

Finally, the protective effect of food matrices should also be considered. Some foods, rich in starch and fiber, provide physical protection for microorganisms during gastric transit. Water‐retaining, fibrous structures, such as boiled potatoes and bread crumbs, are examples of these. Regarding dairy products, buffering foods such as cheese and milk are considered to have the potential to temporarily raise pH and create a protective environment around microorganisms (Corcoran et al. 2007; Jay et al. 2005). These foods are known to act as biological coats in acidic environments, facilitating bacterial survival and gastric passage (Corcoran et al. 2007; Jay et al. 2005). This mechanism should be evaluated, particularly in terms of food‐borne *Sgg* transmission.

### 3.3 Salt Tolerance of *Sgg* at Varying Concentrations

As a result of the salt tolerance tests conducted with the *Sgg* I and II strains, it was observed that the *Sgg* I strain was able to grow in environments containing up to 3% NaCl, while the *Sgg* II strain showed growth in environments with up to 4% NaCl. This is noteworthy when compared with the salt levels permitted in widely consumed cheese varieties. According to the Turkish Food Codex Cheese Communiqué, maximum salt content on a dry‐matter basis varies between 3.0% and 6.5%, depending on the cheese type (Communiqué No. 2015/6, 2015). For example, fresh Kashar Cheese may contain up to 3% NaCl, whereas ripened Kashar can reach 4%–4.5%, and brined or white cheeses may contain even higher levels, up to 6.0%–6.5%. The observed tolerance of the *Sgg* strains also suggests that *Sgg* I and II may have a level of salt tolerance compatible with saltier cheese varieties. Although cheese matrices may present additional inhibitory factors such as lower pH and reduced water activity, these findings indicate that some *Sgg* strains may possess sufficient salt resistance to survive in dairy products.

Beck et al. (2008) reported that 26% (15/58) *Sgg* strains could grow in medium containing 6.5% NaCl. This suggests that approximately one in four strains is capable of growth at high salt concentrations. It should be noted that growth conditions such as medium composition and incubation time differed between our experiments and those reported by Beck et al. (2008), which may partly explain variations in observed salt tolerance.

While salt is an inhibitory environmental stress factor for many microorganisms, it helps some bacteria maintain intracellular osmotic balance by increasing the synthesis of molecules that can act as osmoprotectants (e.g., proline, trehalose, glycine betaine). Thanks to these molecules, bacteria can maintain cell volume and viability in hyperosmotic environments. It has also been reported that bacteria tolerant to environmental stress factors such as *Sgg* can survive in such environments by activating an osmotic stress response (Wood et al. 2001).

### 3.4 Behavior of *Sgg* During Processing and Storage of Kashar Cheese

The effects of different acidification and fermentation approaches on the viability of *Sgg* during Kashar Cheese production showed clear variations among the groups (Table 1).

**TABLE 1:** Mean microbial counts and standard errors detected on M17 Agar at each sampling time in Groups A, B, and C −log_10_ (cfu/g or cfu/mL) and MALDI‐TOF‐MS results of colonies taken at different sampling times.

Sampling times	A		B		C	
	Bacteria grown on M17 (*x̄* ± SEM)	MALDI‐TOF‐MS positives colonies	Bacteria grown on M17 (*x̄* ± SEM)	MALDI‐TOF‐MS positives colonies	Bacteria grown on M17 (*x̄* ± SEM)	MALDI‐TOF‐MS positives colonies
(a) Time of inoculation	8.360^b^ ±0.183	7/9 (78%)	6.376^b^ ±0.101	5/9 (56%)	8.227^c^ ±0.312	1/9 (11%)
(b) Before curd shearing	7.956^b^ ±0.198	3/9 (33%)	5.856^bc^ ± 0.595	3/9 (33%)	9.223^b^ ± 0.359	0/9 (0%)
(c) Curd before scalding (pH: 5)	10.107^a^ ± 0.38	1/9 (11%)	8.287^a^ ± 0.285	1/9 (11%)^†^	10.773^a^ ± 0.24	0/9 (0%)
(d) After scalding	5.089^d^ ± 0.165	3/9 (33%)	2.301^e^ ± 0.174	3/9 (33%)	4.392^d^ ± 0.378	0/9 (0%)
(e) Day 1 (before packaging)	5.159^d^ ± 0.805	1/9 (11%)	2.774^de^ ± 0.386	2/9 (22%)	4.026^d^ ± 0.789	1/9 (11%)
(f) Day 7	6.039^c^ ± 1.011	1/9 (11%)^†^	2.634^de^ ± 0.318	3/9 (33%)	3.994^d^ ± 0.742	0/9 (0%)^†^
(g) Day 15	6.327^c^ ± 1.134	3/9 (33%)	3.467^cd^ ± 0.189	0/9 (0%)^†^	4.287^d^ ± 0.800	0/9 (0%)
(h) Day 30	7.311^b^ ± 0.923	1/9 (11%)	2.201^e^ ± 0.100	2/9 (22%)	4.114^d^ ± 1.081	0/9 (0%)

*Note:* Group A: Acidified with lactic acid alone (*Sgg* 10^8^ cfu/mL); Group B: Acidified with lactic acid alone (*Sgg* 10^6^ cfu/mL); Group C: Acidified with starter culture lactic acid bacteria (*Sgg* 10^8^ cfu/mL). Different lowercase superscript letters (a–e) within the same column indicate statistically significant differences between sampling times for that specific group (*p* < 0.05).

Abbreviations: cfu, colony‐forming unit; MALDI‐TOF‐MS, matrix‐assisted laser desorption/ionization‐time of flight mass spectrometry (used for colony confirmation); SEM, standard error of the mean.

^†^ According to PCR results from colonies selected at these points, one colony in each group tested negative for *Sgg* by MALDI‐TOF‐MS but was positive by PCR. These colonies were excluded from the statistical analysis of MALDI‐TOF‐MS results. Positives colonies indicate verified *Sgg* isolates over total tested colonies.

While no significant growth occurred in Groups A and B during the approximately 90‐min period from inoculation to the curd shearing stage, a tenfold increase was observed in Group C. This difference is attributed to the synergistic effect of the starter culture LAB in Group C, which promotes microbial proliferation, particularly during the curd formation process. The presence of *Streptococcus thermophilus* , which is capable of more rapid growth in symbiosis with *Lactobacillus delbrueckii* subsp. *bulgaricus*, appears to have further supported this increase (Mchiouer et al. 2017).

Microbial growth within the 3‐h phase after curd formation, which encompassed the pressing (A, B, C) and (C) fermentation stages, was substantial in all groups. Between curd shearing and the end of the pressing stage, *Sgg* increased by 2.15 and 3.03 log_10_ in Groups A and B, respectively, whereas a 1.55 log_10_ increase was detected in Group C. Although the microbial growth rate accelerated after curd shearing in Groups A and B, the increase in Group C progressed more steadily throughout the fermentation stage.

Traditionally produced from raw milk, Kashar Cheese is a pasta‐filata cheese widely manufactured in the Balkans and Türkiye. Due to its production characteristics, a 5‐min scalding step at 75°C is considered critical for reducing pathogenic microorganisms. However, previous studies have shown that the whey matrix may limit heat transfer, preventing complete inactivation of some microorganisms during this step (Cetinkaya 2000). Consistent with these findings, the scalding process in our study did not provide complete microbial elimination in any group. A significant suppressive effect on *Sgg* counts was observed in all groups. Upon closer examination, a decrease of 5.02, 5.99, and 6.38 log_10_ in Groups A, B, and C, respectively, demonstrates the average elimination capacity of the scalding step. However, no statistically significant difference was detected in the comparison of percentage change rates between the sampling times at inoculation (a) and after scalding (d) among the groups (*p* = 0.061). The fact that the effect of scalding was 1 log_10_ higher in Group B than in Group A can be attributed to the lower initial concentration in Group B. Meanwhile, Group C exhibited the highest reduction after scalding, consistent with its higher initial bacterial load.

No significant difference was found among the groups in terms of percentage change in microorganism counts between after scalding (d) and day 30 (h) A, B, and C (*p* = 0.097). However, while Groups B and C showed no increase after scalding, Group A exhibited a 2.2 log_10_ rise during refrigerated storage. *Sgg*, which can survive at +4°C during storage, is a bacterium that can adapt to heat and competitive environments (Rusniok et al. 2010). It also maintained its viability in the presence of osmotic stress in brine. These results suggest that traditional cheese production processes may be insufficient to completely eliminate contaminants such as *Sgg*, which can survive measurably in Kashar Cheese for 30 days.

When the groups were evaluated end‐to‐end, the overall reduction in microorganism counts between time of inoculation (a) and day 30 (h) was greater in Group B compared to Groups A and C. A decrease of 4.18 log_10_ occurred in Group B, while reductions of 1.05 and 4.11 log_10_ were recorded in Groups A and C, respectively. The fact that this effect was more pronounced in Group B, with a low inoculation rate, once again demonstrates that the initial contamination load is critical in determining the public health risk. In Groups A and C, where the initial inoculation loads were standardized at the same level (10^8^ cfu/mL), the loss of viability was numerically higher in Group C compared to Group A. Acidification with LAB in Group C may have induced competitive interactions between *Sgg* and LAB, accompanied by the production of bioprotective metabolites (e.g., bacteriocins) (Zacharof and Lovitt 2012). Numerous studies have reported that LAB suppress pathogens through both pH lowering and antimicrobial metabolite production (Gänzle 2015). This may suggest that LAB enhance *Sgg* inhibition. In contrast, acidification with lactic acid alone revealed limited microbial inhibitory potency in Group A despite high inoculation levels.

Exposure to such pathogens can be critical for individuals in at risk groups (e.g., immunocompromised individuals, the elderly, newborns, patients undergoing cancer treatment, and pregnant women) (Jans and Boleij 2018). Selecting starter cultures used in production that not only form curd but also possess bioprotective properties can play an important role in suppressing bacteria such as *Sgg* (Gänzle 2015; Zacharof and Lovitt 2012). Conventional production conditions reduce the viability of *Sgg* but cannot completely eliminate it. The possibility of transmission of zoonotic bacteria through the food chain persists, especially under low‐temperature ripening conditions (4°C). In this context, the protective potential of starter cultures and standardization of heat treatments are important for both product safety and public health.

### 3.5 Preliminary Identification of *Sgg* Colonies With MALDI‐TOF‐MS

MALDI‐TOF‐MS is widely used for microbial identification due to its rapid and accurate performance, with previous studies reporting that scores ≥ 1.80 provide reliable species‐level identification (Huynh et al. 2022; Rosa et al. 2022); thus, in this study, colonies yielding MALDI‐TOF‐MS identification scores of ≥ 1.80 at the species level were considered positive.

In our experimental study, the fact that M17 medium used for the determination of *Sgg* by MALDI‐TOF‐MS is not a selective medium for SBSEC, and that there is currently no selective medium for SBSEC or *Sgg*, is an important factor limiting the analysis results. This medium does not allow selective growth of *Sgg*; other LAB may also develop with similar morphologies. To minimize this limitation, increasing the number of colonies included in the MALDI‐TOF‐MS analysis is recommended, since analyzing more colonies would reduce the effect of random selection and increase the probability of detecting true *Sgg* colonies. To address this limitation, simple preliminary identification methods such as Gram staining, catalase testing, or esculin hydrolysis may be useful for obtaining more definitive results in colony selection. Nevertheless, these methods have important limitations. First, the biochemical features that reliably distinguish *Sgg* from other SBSEC members are very limited. For example, esculin hydrolysis and catalase negativity are shared by most streptococci, making them nondiscriminatory. In addition, *Sgg* shares many overlapping phenotypic traits (e.g., carbohydrate fermentation profiles) with closely related species such as *Sgp* and *Sgm*. This high degree of biochemical similarity often leads to ambiguous or misleading results, showing that phenotypic tests alone are insufficient and molecular confirmation is indispensable for accurate identification (Putnam and Youn 2023; Schlegel et al. 2003).

Detection rates of *Sgg* were evaluated within each group across sampling times using the Friedman test. No statistically significant differences were detected within Groups A, B, or C (A: *p* = 0.384; B: *p* = 0.169; C: *p* = 0.429), indicating that MALDI‐TOF‐MS‐based identification remained relatively stable throughout the production and storage phases. Between‐group comparisons at individual sampling times were performed using the Kruskal–Wallis test. No significant differences were observed after inoculation (a; *p* = 0.100), before curd shearing (b; *p* = 0.350), or before scalding (c; *p* = 0.264). A significant difference emerged only after scalding (d; *p* = 0.030), and post hoc analysis identified this difference between Groups B and C (*p* = 0.037). This point‐specific variation may reflect differences in initial inoculum (lower in Group B) and microbial competition due to LAB activity in Group C. As significance was limited to a single sampling time, this outcome should be interpreted cautiously.

The quantitative distribution of MALDI‐TOF‐MS identifications strongly supports this competitive dynamic. Out of 216 total analyzed colonies, *Sgg* was confirmed in only 20, 19, and 2 colonies in series A, B, and C, respectively, representing an overall detection rate of approximately 19%. Crucially, in Series C where *Streptococcus thermophilus* was introduced as a starter culture, this rate dropped dramatically, with only two out of 72 colonies, corresponding to < 3%, identified as *Sgg*, while the vast majority of the remaining colonies were confirmed as *Streptococcus thermophilus* . Regarding the remaining approximately 81% of the total analyzed colonies across all series, they were predominantly identified as typical background LAB and environmental microflora originating from the traditional cheesemaking process; thus, their detailed individual profiles were excluded to maintain the primary focus on the target pathogen. This minimal recovery rate can be interpreted through two distinct microbiological scenarios. First, it may provide robust biological evidence that the rapid proliferation and acidogenesis of the yogurt starter culture successfully outcompeted and suppressed the clinical *Sgg* strains within the cheese matrix. Alternatively, given that *Sgg* and *Streptococcus thermophilus* exhibit nearly identical colony morphologies on M17 agar, this outcome may reflect a statistical dilution effect rather than absolute inhibition. Because the starter bacteria reach overwhelming dominance during fermentation in Group C, the probability of randomly picking a true *Sgg* colony among thousands of morphologically indistinguishable target colonies becomes exceptionally low. Overall, this critical limitation should be taken into account when discussing the evolution of the microbial counts, as the high populations visualized on the nonselective agar plates during ripening and storage primarily reflect the overgrowth of background starter microbiota rather than the target pathogen.

Therefore, for each sampling time, three colonies were randomly selected for MALDI‐TOF‐MS analysis. This limits the probability of detecting true *Sgg* colonies, especially at stages where their proportion within the overall microbiota is expected to be low due to scalding stress, acidification, and competitive pressure from rapidly growing LAB. In this context, the detection of *Sgg* at several sampling times, even when only three colonies were analyzed, is noteworthy and suggests that the organism may persist at low abundance within the cheese matrix despite processing stresses. The intermittent absence of detection can be attributed to the limited number of colonies screened rather than a complete elimination of the organism. These findings indicate that fluctuations in detection rates are influenced not only by biological survival but also by the randomness of colony selection from a nonselective medium.

Among the species within the SBSEC group *Sgg, Sgp*, and *Sgm* cannot be fully distinguished from each other in MALDI‐TOF‐MS databases due to their similar protein profiles (Putnam and Youn 2023; Schlegel et al. 2003). While this provides high selectivity at the species level, it necessitates supporting subspecies identifications with molecular methods (Kim et al. 2024). In our study, many colonies were identified as *Sgp* subtype. The protein profile of this subtype is highly similar to *Sgg*; therefore, it is possible that misclassifications at the subspecies level may occur in MALDI‐TOF‐MS analyses (Pan et al. 2023). These results suggest that a limited number of true Sgg may be present in colonies pre‐identified by MALDI‐TOF‐MS, thereby demonstrating the importance of molecular confirmation for accurate identification at the subspecies level. Future work should focus on developing selective media or expanding MALDI‐TOF‐MS databases with well‐characterized reference strains.

### 3.6 PCR Confirmation

To compare the results of MALDI‐TOF‐MS with PCR, 10 colonies were selected. By MALDI‐TOF‐MS, three of them (dB2‐3, aA1‐3, eA2‐1) were positive for *Sgg* while the other five (gB1‐1, hC1‐2, fC2‐2, cB3‐3, fA1‐2) were among the *Sgg*'s close relatives within the SBSEC group. Additionally, two colonies (dB3‐1, eA3‐1) identified as belonging to a different genus were included as negative controls (Table 2).

**TABLE 2:** Evaluation of MALDI‐TOF‐MS and PCR results for selected colonies.

Colony code	Positive control		Negative control (Master mix)	gB1‐1	hC1‐2	fC2‐2	dB2‐3	aA1‐3	eA2‐1	cB3‐3	dB3‐1	fA1‐2	eA3‐1
	*Sgg* I	*Sgg* II											
Closest result with MALDI‐TOF‐MS	*Sgg*	*Sgg*	nd	*Sgp*	*Sgp*	*Sgp*	*Sgg*	*Sgg*	*Sgg*	*S. equinus*	*Micrococcus luteus*	*Sgp*	*Enterococcus durans*
PCR confirmation for *Sgg*	+	+	−	+	−	+	+	+	+	+	−	+	−

*Note:* While colony **cB3‐3** was initially identified as *S. equinus* , and colonies **fC2‐2** and **fA1‐2** as *Sgp* via MALDI‐TOF‐MS, all three were definitively confirmed as *Sgg* by PCR.

Abbreviations: −, no amplification in at least one discriminatory locus; +, positive amplification in all seven *Sgg*‐specific loci; nd, not detected; *Sgg*, S. *gallolyticus* ssp. g*allolyticus*; *Sgp*, *S. gallolyticus* ssp. pasteurianus.

**FIGURE 3:**
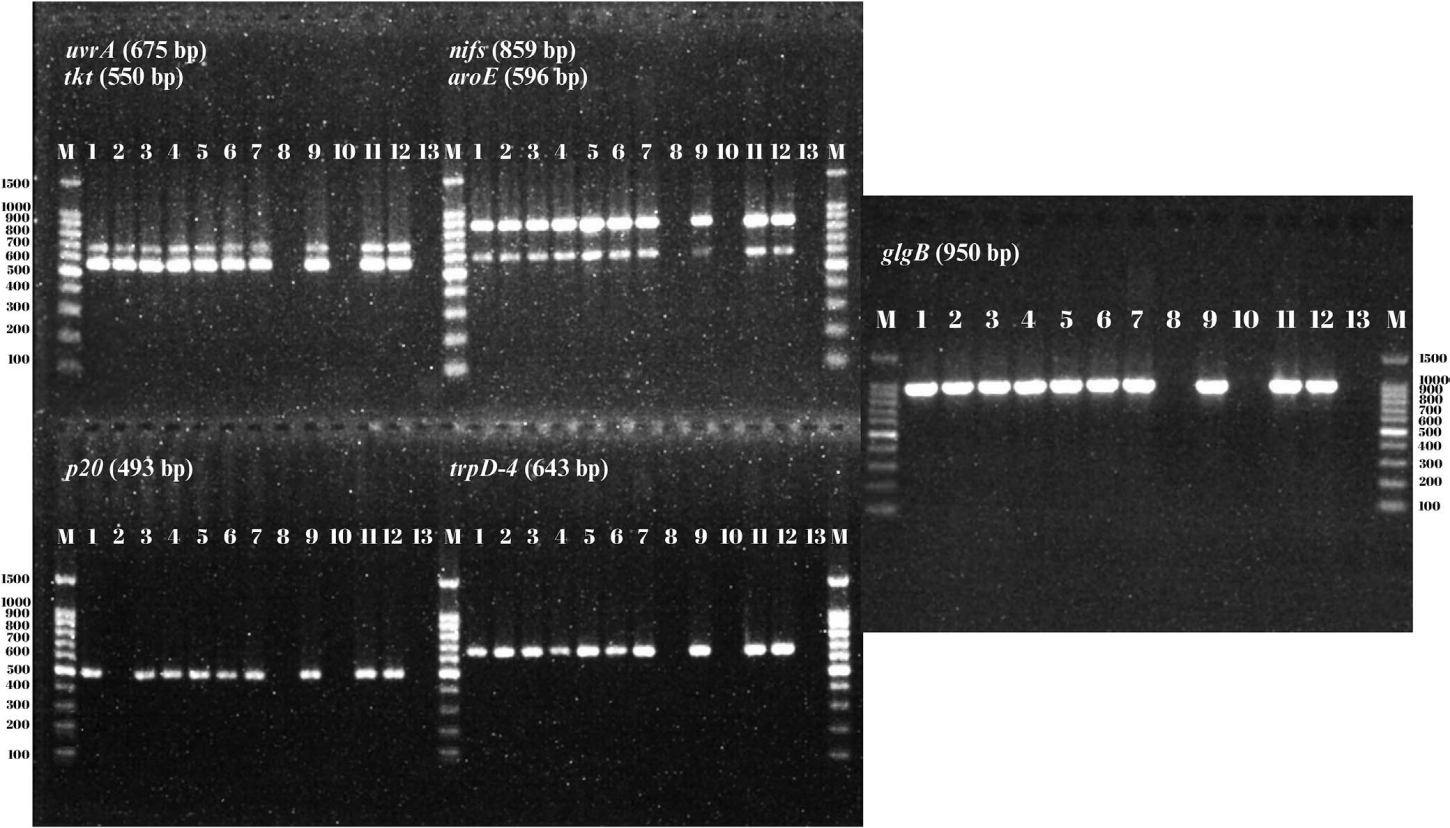
Agarose gel electrophoresis showing PCR amplification products from selected colonies. Seven target genes specific *for Streptococcus gallolyticus* subsp. *gallolyticus* (uv*rA* 675 bp, *tkt* 550 bp, *nifS* 859 bp, *aroE* 596 bp, *p20* 493 bp, *trpD*‐4643 bp, *glgB* 950 bp) were amplified. Lanes M: DNA ladder; Lanes 1–10: Selected colonies (gB1‐1, hC1‐2, fC2‐2, dB2‐3, aA1‐3, eA2‐1, cB3‐3, dB3‐1, fA1‐2, eA3‐1); Lanes 11–12: Positive controls (*Sgg* I, *Sgg* II); Lane 13: Negative control (master mix only).

Positive amplification results were achieved in all seven gene regions of all streptococci (gB1‐1, fC2‐2, dB2‐3, aA1‐3, eA2‐1, cB3‐3, fA1‐2), except for colony hC1‐2, which was identified as *Sgp* based on MALDI‐TOF‐MS scores. This result indicates that colony hC1‐2 was only partially positive in molecular confirmation with *Sgg*‐specific primers. However, since the *p20* gene played a discriminatory role, this colony could be considered negative for *Sgg*; however, more detailed studies are necessary to provide evidence for the correct identification of this colony. The colony cB3‐3, identified as *S.equinus* by MALDI‐TOF‐MS, gave bands in all seven gene regions and was confirmed to be *Sgg*. Similarly, colonies gB1‐1, fC2‐2, and fA1‐2, which were initially classified as *Sgp* by MALDI‐TOF‐MS profiling, also yielded positive bands across all seven gene targets and were definitively confirmed to be *Sgg* via PCR (Figure 3). These results clearly demonstrate that MALDI‐TOF‐MS is a powerful and reliable technique; however, for SBSEC members, it has some limitations, especially at the subspecies level. Therefore, an *Sgg*‐specific molecular confirmation approach, such as PCR, would increase the reliability of identification and subspecies discrimination (Putnam and Youn 2023). The PCR protocol applied in this study offered a strong alternative in terms of specificity, enabling the detection of both false positives and interspecies transitions. Consequently, it has been emphasized that integrated molecular confirmation methods are critical for reliable identification of complex taxonomic groups such as SBSEC members in food, clinical and environmental samples.

### 3.7 Physicochemical Changes During Cheese Processing and Storage

The results of the physicochemical analysis are presented in Table 3. When the behavior of *Sgg* was evaluated in relation to the basic chemical parameters of fresh Kashar Cheese, only the values obtained at the *h* sampling point (final product) were statistically compared among Groups A, B, and C. The analysis revealed that pH values (*p* = 0.066), titratable acidity (*p* = 0.051), and water activity (*p* = 0.046) did not differ significantly between the groups after post hoc correction. Similarly, moisture, dry matter, salt, and fat contents showed no significant differences after post hoc correction, despite the initial global variance (*p* = 0.027). These results indicate that none of the major chemical components of the cheeses were influenced by either the microbial load (initial inoculation concentration) or the acidity control method (lactic acid or yogurt cultures) at the final product stage.

**TABLE 3:** Pooled physicochemical results of fresh Kashar cheese (A_1‐2‐3_, B_1‐2‐3_, C_1‐2‐3_) (mean ± SEM).

Sampling times	pH values	Titratable acidity values (% LA)	Water activity values (*a*_*w*_)	Moisture content (%)	Dry matter content (%)	Salt content in dry matter (%)	Fat content in dry matter (%)
(a) Time of inoculation	6.63 ± 0.12	0.106 ± 0.01	—	—	—	—	—
(b) Before curd shearing	5.41 ± 0.41	0.244 ± 0.07	—	—	—	—	—
(c) Curd before scalding	5.28 ± 0.07	0.384 ± 0.02	0.989 ± 0.003	48.99 ± 0.84	51.01 ± 0.84	—	44.94 ± 0.67
(d) After scalding	5.31 ± 0.06	0.584 ± 0.02	0.982 ± 0.002	45.25 ± 0.87	54.75 ± 0.87	2.376 ± 0.14	42.48 ± 0.62
(e) Day 1	5.31 ± 0.06	0.800 ± 0.00	0.963 ± 0.001	45.05 ± 0.91	54.95 ± 0.91	2.319 ± 0.18	43.22 ± 0.74
(f) Day 7	5.38 ± 0.08	0.908 ± 0.05	0.963 ± 0.003	43.80 ± 1.22	56.20 ± 1.22	1.996 ± 0.20	42.88 ± 0.86
(g) Day 15	5.38 ± 0.08	1.032 ± 0.05	0.956 ± 0.007	41.96 ± 0.99	58.04 ± 0.99	1.751 ± 0.16	41.60 ± 0.76
(h) Day 30	5.39 ± 0.08	1.144 ± 0.03	0.957 ± 0.007	41.66 ± 0.93	58.34 ± 0.93	1.523 ± 0.26	41.49 ± 0.61

*Note:* Each value represents the mean ± standard error of the mean (SEM) of the experimental groups. Emdash (–) indicates that the parameter was not analyzed or not applicable at that specific processing stage. Moisture, dry matter, salt, and fat contents are expressed as percentages (%). Salt and fat contents are calculated and presented on a dry matter basis.

Abbreviations: % LA, percentage of lactic acid equivalent; *a*_*w*_, water activity.

Importantly, in all three groups, the salt content was below the upper limit of 3% salt in dry matter defined in the Turkish Food Codex Cheese Communiqué (Communiqué No. 2015/6, 2015) for fresh Kashar Cheese, demonstrating compliance with the quality standards.

Although no statistically significant differences were found, biological acidification with yogurt cultures in Group C appeared to promote both microbial inhibition and a more balanced physicochemical profile of the cheese. In contrast, Group A, where acidity was adjusted chemically with lactic acid, tended to have a softer texture but exhibited higher vulnerability to pathogen survival due to the lack of competitive inhibition and antimicrobial metabolites typically provided by live starter cultures. Group B represented a transitional product in terms of both structure and components, offering moderate stability. Previous studies have similarly reported that LAB‐driven acidification contributes to microbial suppression and improved stability, even in the absence of strong compositional changes (Gänzle 2015; Zacharof and Lovitt 2012).

Ultimately, product quality and safety are determined not only by pH or acidity but also by the interaction of multiple factors, including acidification method, microbial presence, salt levels, and cheese matrix structure.

## 4 Conclusion

This study is, to our knowledge, the first comprehensive study to experimentally investigate the viability and behavior of *Sgg*, a potentially zoonotic and opportunistic food pathogen, during the production and storage of fresh Kashar Cheese. Using two clinical strains isolated from patients with IE and CRC, the study demonstrated that *Sgg* remained viable at different stages of the production process and was detectable in the final product.

Among different acidification strategies, fermentative acidification using yogurt culture was found to be more effective in reducing *Sgg* viability. This was likely due to the suppressive effect of yogurt bacteria and the competitive overgrowth of *Streptococcus thermophilus* , which was clearly reflected in the low confirmation rate of *Sgg* (< 3%) via MALDI‐TOF‐MS in these batches. In contrast, artificial acidification using lactic acid maintained the bacterium's viability for longer periods. While the scalding process at 75°C significantly reduced the microbial load on M17 agar, viable *Sgg* strains were still isolated and confirmed in some batches after this process, suggesting that the applied thermal treatment may not be fully effective under all processing conditions. The findings demonstrate that despite being heavily outnumbered by background and starter microbiota, clinical *Sgg* strains possess sufficient acid and thermal tolerance to survive in fresh Kashar Cheese throughout a 30‐day storage period. Consequently, the risk of foodborne transmission cannot be ruled out. Given *Sgg*'s known association with serious systemic diseases, its ability to persist in dairy matrices is of critical importance for both food safety and public health. This concern is particularly acute for traditional cheeses produced from raw milk, where the absence of controlled pasteurization elevates the risk of pathogen survival. Therefore, strict hygienic milking, validated heat treatments, the use of protective cultures, and strengthened food safety management systems are essential to control the dissemination of SBSEC members. In conclusion, this study raises awareness of the risks of potential zoonotic transmission from dairy products and highlights the critical need to reassess traditional food safety practices. Moving forward, the findings of this work open important avenues for future research. First, whole‐genome sequencing of these specific clinical *Sgg* strains is heavily warranted to fully characterize their virulence factors, antibiotic resistance profiles, and specific genetic determinants driving their high thermal and acid tolerance during cheese production. Furthermore, since bacterial behavior is highly dependent on the food matrix, future studies should investigate the survival and persistence of these strains in diverse cheese varieties (such as hard, long‐ripened cheeses or high‐moisture fresh cheeses) to establish comprehensive risk models across different dairy ecosystems.

Ultimately, these perspectives show that we need to improve routine food safety tests by using modern molecular and proteomic tools, such as qPCR and MALDI‐TOF‐MS, in dairy production. These advanced methods are necessary to avoid the mistakes of traditional, nonselective agar media, ensuring we do not underestimate or overestimate opportunistic pathogens in fermented foods.

Furthermore, these findings open the door for new food protection strategies. Future research should focus on finding and developing protective starter cultures that can produce specific natural antimicrobials (bacteriocins) to stop the growth of the SBSEC complex. Using these new testing and biocontrol methods will help protect traditional dairy products, improve official food safety rules, and reduce the public health risks of animal‐to‐human disease transmission through food.

## Author Contributions

**Cüneyt Özakın:** conceptualization, formal analysis, writing – review and editing. **Artun Yıbar:** conceptualization, methodology, data curation, investigation, validation, writing – review and editing. **Nazmiye Ülkü Tüzemen:** conceptualization, formal analysis, writing – original draft, writing – review and editing. **Müge Kayapınar:** conceptualization, software, data curation, validation, formal analysis, visualization, writing – original draft, writing – review and editing. **Recep Çıbık:** conceptualization, methodology, software, data curation, investigation, validation, formal analysis, supervision, funding acquisition, visualization, project administration, resources, writing – original draft, writing – review and editing.

## Funding

This work was supported by the Scientific Research Projects Coordination Unit of Bursa Uludag University (Project No: TOA‐2022–1137), within the scope of the Priority Area Research Projects Program.

## Conflicts of Interest

The authors declare no conflicts of interest.

## Data Availability

The data that support the findings of this study are available from the corresponding author upon reasonable request.
